# Twenty Years after Bovine Vaccinia in Brazil: Where We Are and Where Are We Going?

**DOI:** 10.3390/pathogens10040406

**Published:** 2021-03-31

**Authors:** Iago José da Silva Domingos, Jaqueline Silva de Oliveira, Kamila Lorene Soares Rocha, Danilo Bretas de Oliveira, Erna Geessien Kroon, Galileu Barbosa Costa, Giliane de Souza Trindade

**Affiliations:** 1 Laboratório de Vírus, Departamento de Microbiologia, Instituto de Ciências Biológicas, Universidade Federal de Minas Gerais, Belo Horizonte, MG 31270-901, Brazil; iago.jsd@gmail.com (I.J.d.S.D.); jaquelinebmedica@hotmail.com (J.S.d.O.); kroone@icb.ufmg.br (E.G.K.); 2 Laboratório de Doenças Infecciosas e Parasitárias, Faculdade de Medicina, Universidade Federal dos Vales do Jequitinhonha e Mucuri, Diamantina, MG 39100-000, Brazil; kamilalsr@yahoo.com.br (K.L.S.R.); danilo.bretas@ufvjm.edu.br (D.B.d.O.); 3 Departamento de Análise em Saúde e Vigilância de Doenças Não-Transmissíveis, Secretaria de Vigilância em Saúde, Ministério da Saúde, Brasília, DF 70719-040, Brazil

**Keywords:** *Vaccinia virus*, bovine vaccinia, public health, zoonosis, neglected disease, laboratory diagnosis

## Abstract

Orthopoxvirus (OPV) infections have been present in human life for hundreds of years. It is known that Variola virus (VARV) killed over 300 million people in the past; however, it had an end thanks to the physician Edward Jenner (who developed the first vaccine in history) and also thanks to a massive vaccination program in the 20th century all over the world. Although the first vaccine was created using the Cowpox virus (CPXV), it turned out later that the Vaccinia virus was the one used during the vaccination program. VACV is the etiological agent of bovine vaccinia (BV), a zoonotic disease that has emerged in Brazil and South America in the last 20 years. BV has a great impact on local dairy economies and is also a burden to public health. In this review, we described the main events related to VACV and BV emergence in Brazil and South America, the increase of related scientific studies, and the issues that science, human and animal medicine are going to face if we do not be on guard to this virus and its disease.

## 1. Introduction

For centuries in world history, humanity has faced a fatal disease named smallpox, which seemed impossible to fight. Throughout its existence, smallpox was responsible for the death of hundreds of millions of people worldwide. Its eradication in 1980 is considered one of the most outstanding achievements of human medicine and public health, reaching its 40th anniversary in 2020 [1,2]. The elimination of smallpox was only possible thanks to an eradication program developed and adopted for decades by the World Health Organization (WHO), which consisted of an extensive vaccination of the entire world population in the 20th century. After smallpox was declared eradicated, there was no need to keep vaccinating people once the Variola virus (VARV) was no longer circulating among the human population. Therefore, the worldwide vaccination was discontinued [3].

Historically, vaccinology started with the smallpox vaccination process developed by the British physician Edward Jenner at the end of the 18th century [4]. Jenner curiously observed that the injuries caused by the Cowpox virus (CPXV) in humans, due to the direct contact with infected cows during the milking process, were similar to the lesions caused by VARV [1,4,5,6]. Hence, the observation of CPXV infections was the preamble to the first vaccine produced worldwide.

Jenner used samples of CPXV collected from infected dairy cows, inoculated in humans, and found that these individuals did not develop smallpox later, confirming the hypothesis of cross-immunization between viruses belonging to the genus *Orthopoxvirus* (OPV) [7]. From this discovery, the technique was improved over the following years and gave rise to the first vaccine in history, which was distributed around the world through arm-to-arm transport, and a few years later would be produced by experimentally infected calves [1,4,6].

After several decades of vaccination, the CPXV isolate used in this vaccine distributed worldwide was supplanted by Vaccinia virus (VACV) [8]. The exact moment in history that it occurred is still unknown, but since the studies about this virus kept increasing throughout the years, VACV was used instead of CPXV in the 20th century in the WHO eradication program of VARV [9]. One of the exciting and useful features of the OPV is sharing an immunological cross-reactivity due to the antigenic similarity among its species [7,10]. The use of VACV and its effectiveness in the eradication of smallpox has made this virus extremely important in the history of virology and immunology. However, even presenting a tremendous scientific relevance and diversity of research on VACV worldwide, its origin and natural reservoir are still unknown [1]. In Brazil, in the last 20 years, there are records of the emergence of a disease directly related to VACV, the bovine vaccinia (BV), which causes natural infections in cattle herds and humans [11,12,13].

BV is an emerging viral zoonosis characterized by ulcerative lesions on the skin and mucous membranes, affecting mainly dairy cattle and milkers [14]. In dairy cattle, the lesions occur mostly on the teats and udders, are often accompanied by mastitis that can progress to the temporary commitment of the mammary glands. Consequently, it decreases milk production, causing a significant impact on the dairy economy, reducing profits [1,14,15,16,17].

In humans, the clinical manifestations of BV are mainly lesions located at the primary site of infection, usually on the fingers and hands of milkers who have a history of unprotected contact with infected animals [12,14,15,17]. However, additional lesions as a result of the self-inoculation process have also been described in other body sites such as the face, eyes, and genital region, and in humans, BV is associated with high morbidity [18,19,20,21]. The process between the onset of the initial symptoms and the healing of the ulcerative skin lesions takes approximately 21 days. Systemic symptoms such as fever, headache, malaise, myalgia, inguinal, and cervical lymphadenopathy are also present about three days after the initial symptoms [16,17,18,19,20].

The classic form of VACV transmission is through direct contact between the milkers and infected dairy cows, which characterizes BV as an occupational zoonosis [16]. Furthermore, the sick dairy workers are often removed from work, which results in disruption of the service in the affected rural properties, as well as additional expenses related to the replacement of human resources and decreases the familiar incomes of these affected milkers [13,20,22,23]. The following discussion of VACV and BV researches illustrates chronologically how they have become a very important part of virology and epidemiology history in Brazil during the past 20 years.

## 2. The Main Areas of BR-VACV Endemicity and the Epidemiological Profile

**Figure 1:**
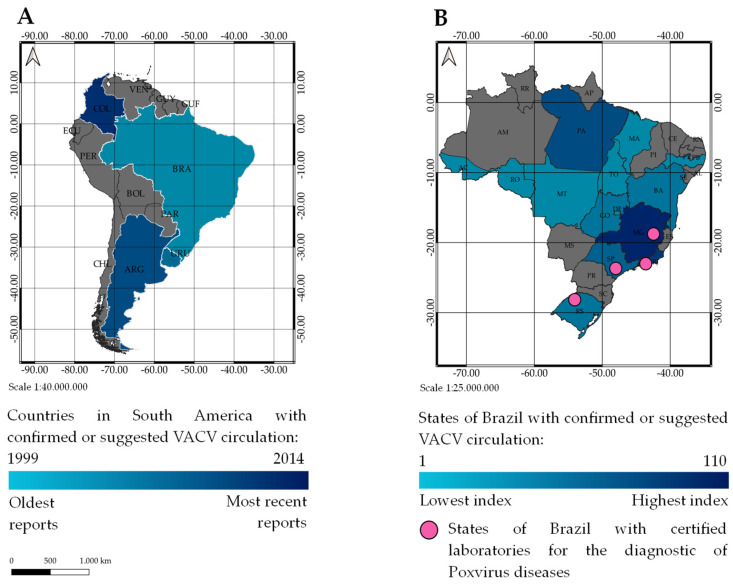
Characterization of vaccinia virus circulation in the South American continent. (**A**) Map of South America indicating the countries where VACV has been detected or suggested to be circulating. The countries in gray color have not recorded VACV detection or suggestion so far. The blue gradient in the left highlights the first records of VACV in Brazil starting in 1999 to the last one in 2014, in Colombia. (**B**) Map of Brazil indicating the States where VACV has been detected or suggested to be circulating. The states in gray color have not recorded VACV detection or suggestion so far. The blue gradient in the right highlights the States from the lowest to the highest index of records reported by scientific publications. Acre (AC), Maranhão (MA), Mato Grosso (MT), Pernambuco (PE), Rondônia (RO), Tocantins (TO), and the Federal District (DF) present only one record each. On the other hand, Minas Gerais (MG) is the state with the highest number of VACV cases (110 records). The pink circles indicate the States of Brazil where there are certified laboratories for the diagnosis of Poxvirus diseases. This map was made using the Free and Open Source QGIS program based on free shapefiles by Instituto Brasileiro de Geografia e Estatística (IBGE) available at https://www.ibge.gov.br/geociencias/downloads-geociencias.html (accessed on 16 October 2020).

The first BV outbreaks were identified in the Southeastern region of Brazil, in the States of São Paulo and Rio de Janeiro in 1999, followed by Minas Gerais in 2000 [11,12]. After the initial records of BV outbreaks, VACV has quickly spread to other states and reported in all Brazilian territory. In the past few years, the occurrence of VACV has been reported for the first time in history in Brazil’s neighboring countries. The current epidemiological scenario shows the circulation of VACV in an extensive area of the Brazilian territory and other South American countries such as Argentina, Uruguay, and Colombia (Figure 1) [17,24,25,26,27,28].

Outbreaks and/or VACV occurrences have been recorded in 11 Brazilian states over these 20 years [29,30,31,32,33,34,35,36,37,38,39,40,41,42,43]. However, only in the States of Minas Gerais and Goiás (Midwest region) BV is mandatory reported by the public health departments [44,45]. It is also interesting to note that there is a centralization of the diagnosis in the laboratory response network located in few research centers or governmental institutions. These reference centers are distributed in the States of Minas Gerais, São Paulo, and Rio de Janeiro (Southeast region), and in the State of Rio Grande do Sul (South region). With the increasing number of BV cases over time, this scenario reflects how BV and VACV occurrences are still neglected in Brazil. Therefore, forming a cooperative network with strategic actions to communicate, monitor, prevent, and improve research is very important and recommended. This network would be essential to increase partnerships and investments in health, agriculture, and environment research, for a prompt national and global response to reduce the impacts caused by VACV. However, as suggested by Zanella and co-workers, the surveillance of VACV is very scarce, creating more space for the virus to spread and increasing the burden related to its occurrence [46].

**Figure 2:**
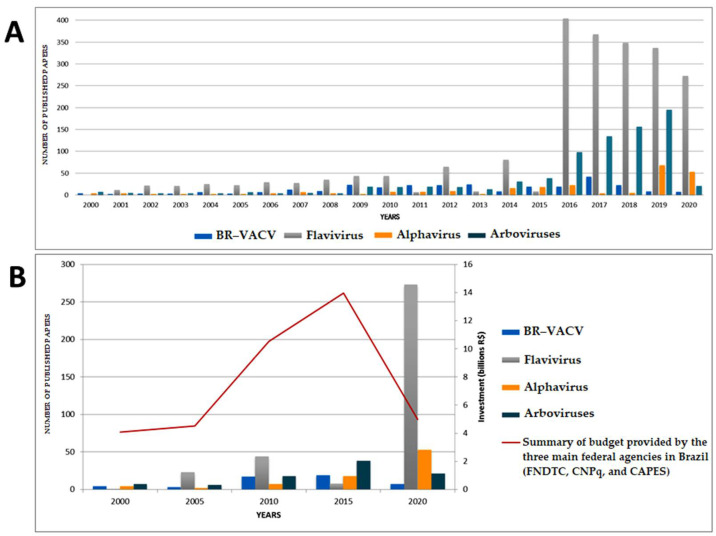
(**A**) Distribution of published scientific papers about Vaccinia virus and/or Bovine Vaccinia, Flavivirus, Alphavirus and Arboviruses during 2000–2020 in Brazil. We included a total of 3.511 publications identified by conducting electronic searches in PubMed platform, available at https://pubmed.ncbi.nlm.nih.gov/ (accessed on 15 March 2021). Published studies were identified using the keywords Vaccinia virus, Bovine Vaccinia, and Brazil (*n* = 281); Flavivirus and Brazil (*n* = 2.184); Alphavirus and Brazil (*n* = 243); and Arboviruses and Brazil (*n* = 803). An average of 13.4 VACV publications per year were identified. (**B**) Analysis between the numbers of published papers in Brazil (according to the theme of interest) and the summary of scientific research investments over the past twenty years. Although the decrease in publications of BR-VACV is related to the reduction in funding for its research, the same is not valid for the research related to arboviruses.

The first signs of the emergence of BV in Brazil prompted numerous relevant questions regarding VACV epidemiology, natural history, its hosts, and transmission chain [12,15,29]. Figure 2 shows the number of scientific publications related to the emergence of VACV in Brazil over the past 20 years and the discoveries regarding its natural history and other related fields. It is possible to observe a gradual increase in scientific publications related to VACV and BV until 2017. From that year on, the number of scientific publications decreased drastically until 2020. It is also important to highlight a massive reduction in the budget destinated to science and public health in Brazil in the last four years [47,48]. Moreover, Brazil has experienced Dengue, Zika, Yellow Fever, and Chikungunya viruses threat, which have set up significant public health issues, compromising the financial support to study other viruses and pathogens of great public health interest.

After the emergence of VACV and the evolution of studies related to its natural circulation, several findings of great relevance have supported its epidemiology. The first studies associated with the seroprevalence of OPV in rural populations emerged in 2010. Mota and colleagues retrospectively analyzed individuals from Amazon rural villages in North Brazil, finding a seroprevalence of 27.9% [49]. Interestingly 23.4% of the individuals were not vaccinated against smallpox, suggesting they could be exposed to naturally circulating OPV [49]. In 2015, a report from Figueiredo et al. showed a seroprevalence of 9.8% in individuals from rural areas where BV cases have not been observed since the late 1990s [50]. The value of seroprevalence studies as surveillance tools for infectious diseases in the general population is significant. Hence, to better understand the burden of VACV infections, to identify risk factors, target interventions, and monitor trends, Costa and colleagues designed the first epidemiological study in an endemic area of Brazil [43]. In that survey, almost 31% of studied individuals tested positive for neutralizing antibodies. Increasing of age and previous BV outbreak in the rural properties were independently associated with neutralizing antibodies [43].

Borges and colleagues described a seroprevalence of neutralizing antibodies against OPV in 75.7% of dairy cows sampled in rural communities of Minas Gerais State [22]. Furthermore, the presence of domestic felids on a property was significantly associated with diminished odds of a cow having OPV-neutralizing antibodies. Another study also conducted in Minas Gerais State revealed a seroprevalence of neutralizing antibodies against OPV in 20.6% was also described in equids, raising questions about the role of equids in the VACV epidemiological chain, as well as unrecognized infections and silent circulation [51].

## 3. Genetic Characterization of Brazilian Vaccinia Viruses (BR-VACV)

Over the past 20 years, the genetic characterization of the BR-VACV isolates is generally carried out by analyses of specific genetic markers. However, the absence of whole-genome sequences from isolates represents a significant gap to better understand the origin and evolutionary history of the viruses circulating in Brazil. Despite that, another significant discovery is the demonstration that BR-VACV is grouped into at least two different clusters based on genetic diversity: group I and group II [52,53,54].

Through histopathological and immunohistochemistry analysis in experimentally infected BALB/c mice, it was possible to identify the virulence patterns presented by different VACV isolates [52,53,54,55,56,57,58]. The isolates belonging to group I are less virulent in vitro and in the murine model, while the isolates belonging to group II exhibit great virulence [53,54,57]. Subsequent findings demonstrated that these two groups could co-circulate in the same geographic area and co-infect the same host [34,52]. Despite the existence of two VACV groups circulating in Brazil, the group I viruses have been more frequently isolated when compared to viruses from group II. However, the available laboratory methodology for isolation could be a bias [55].

The first isolation of two different VACV strains in the same BV outbreak was described among neighboring farms in the rural area of the state of Minas Gerais, in 2001. During this outbreak, the viruses named Guarani P1 virus (GP1V) and Guarani P2 virus (GP2V) were isolated and characterized [52]. It was observed that several conserved genes also present in other representatives of OPV genus were detected in both GP1V and GP2V. However, this study’s main finding was to indicate that different isolates from different places could establish a natural circulation, demonstrating that VACV can have multiple origins and raise a new perspective to explain the genetic diversity observed in Brazil [14,53]. Future studies would reinforce the hypothesis of VACV coinfection in the same host during the same outbreak, showing the existence of a diversity of clones associated with viral infection [54,55,59]. Although these findings are essential in the VACV history, the knowledge regarding how the diversity of clones and genetic variety can interfere in BV outbreaks with different characteristics, different virulence patterns, and clinical presentation in animals and in humans still need to be better explored.

The approach of VACV genetic diversity in Brazil has expanded considerably. Some genes have been identified as useful markers to discriminate between the BR-VACV groups. Analysis of the A56R gene, that encodes for the viral hemagglutinin, revealed a molecular signature based into 18-nucleotide deletion in Group I of BR-VACV [11,12,14]. Another 18-nucleotide deletion in BR-VACV Group I was observed for the gene that encodes the A-type inclusion body protein (A26L), together with an additional 12-nucleotide deletion [60]. Furthermore, a 10-nucleotide deletion is also present for the gene that encodes the CC-chemokine-binding protein (C23L) [57] and a 15-nucleotide deletion for the serine protease inhibitor-3 gene (K2L) gene [54]. On the other hand, these molecular signatures are not detectable in group II, which is composed of several Brazilian isolates and the reference sample VACV–Western Reserve [11,12,14,54,57,60]. These approaches are important, not only for sample identification but also to infer ancestry and to investigate hypothetical correlation of each sample or group with its unique epidemiological and biological features.

Many studies focused on identifying molecular targets in the following years allowed a better characterization of viral diversity. These efforts were also significant for the evolution of the VACV diagnosis, and several protocols for laboratory identification became available [57,58,59,60,61,62,63]. However, the evolution of the viral isolation, identification, and characterization protocols was not followed by its implementation in the country’s reference laboratories, showing a gap between the scientific discoveries and sanitary agencies and responsible organs positioning or animal defense facing outbreak emergencies in Brazilian states.

The complete genome sequences of only two BR-VACV are available so far, Cantagalo and Serro viruses [56,64]. Phylogenetic analysis performed by Medaglia and colleagues showed evidence of a novel, independent cluster of VACV formed by the wild strains Cantagalo and Serro viruses, the Brazilian vaccinal strain IOC (VACV-IOC), and *Horsepox virus* (HSPV). These findings support the hypothesis that BR-VACV could be derived from an ancient smallpox vaccine sample related to *Horsepox virus* that escaped to nature, representing feral VACV that evolved independently of the Brazilian vaccinal strain used in the 1970s after splitting from a most recent common ancestor related to *Horsepox virus* [64]. However, the lack of additional genome sequences of wild BR-VACV samples hampers a conclusive statement about their origins.

Recently, the analysis of the complete genome of clinical isolates of the Cantagalo virus, collected in the early years and at the epicenter of the emergence of VACV in Brazil, revealed genetic characteristics not shared among the isolates. These data suggest different events of VACV spreading in the Southeastern region of Brazil, reflecting the complex genomic diversity of the isolates related to the first outbreaks [65]. The sequencing of more isolates would allow better identification of new genetic markers, elucidates the genetic diversity of BR-VACV that circulate throughout Brazil and probably in other South American countries. The whole-genome sequencing could also help shed light regarding the origins of BR-VACV.

## 4. Classic Transmission and Alternative Routes for Zoonotic BR-VACV Infections 

The first studies related to VACV and BV in Brazil focused on describing outbreaks affecting bovines and humans to understand the transmission dynamic, identification, and biological and molecular characterization of the etiological agent [11,12,14].

After that, the identification of the host spectrum has also been one of the main themes investigated. The detection of VACV in wild rodents, non-human primates, marsupials, procyonids, and equids a few years after the registration of the first BV outbreak has contributed significantly to assessing the dynamics of virus circulation and maintenance in wild and rural environments [30,31,32,33,34,35,36,37,66]. Additionally, VACV circulation has also been detected among domestic animals (cats and dogs) in urban areas [67,68] (Supplementary Table S1).

Although many studies have been trying to elucidate the specific role of farming and wild animals in the VACV transmission chain, there are still many gaps to fill regarding the occurrence of BV outbreaks. However, once VACV was detected in a broad spectrum of hosts in rural and urban environments, it is possible to suggest that some of the particular species can contribute to the spread of VACV to new environments [32,67,68]. Some studies have shown that bovines have a crucial role as viral amplifiers due to the elimination of infectious viral particles through feces and milk in the environment [12,69,70]. These findings suggest that the infectious particles eliminated in the environment could be a source of contamination to other animals (e.g., rodents) in rural areas, spreading to wild areas and maintaining the viral dynamic in nature [33,68,70,71]. Thereby, further studies and the adoption of preventive measures are necessary to reduce the impact of VACV infections and BV outbreaks. Considering the clinical manifestations of BV, if there is no knowledge for correct management during new and frequent outbreaks, it is not difficult to imagine the increasing burden of BV in the near future.

The establishment of protocols for the detection and characterization of circulating BR-VACV provided the expansion of studies related to the viral host spectrum knowledge. Thus, the detection and characterization of BR-VACV in wild and urban environments (besides rural) began to be conducted in Brazil around the decade of 2010. In the study conducted by Abrahão and colleagues, a VACV strain named Mariana virus (MARV) was isolated from a peridomestic rodent during a BV outbreak in a rural area of Minas Gerais State [33]. Additionally, the same virus was also isolated from humans and dairy cattle affected during that outbreak. This fact strengthens the hypothesis that other animals such as rodents, could be potentially related to the spread of VACV from the rural to wild environments and vice versa [33]. Further studies conducted in areas with history of BV outbreaks corroborated Abrahão’s findings, demonstrating the circulation of VACV in wild and synanthropic rodents, reinforcing the hypothesis that wild rodents could be implicated as viral reservoirs [66,72,73].

The studies related to the VACV circulation in wild environments raised epidemiological questions regarding its transmission cycle. Hence, Abrahão et al. (2009) and Miranda et al. (2017) proposed hypothetical models for VACV transmission through different environments [33], and interaction networks highlighting the wild animals as links between natural and anthropic environments [66]. Furthermore, Costa and colleagues have also proposed a hypothetical model to explain the circulation of VACV in domestic dogs and wild coatis from a transitioning area between urban and wild environments [68]. Using a serological and molecular approach, it was found that the animals were exposed to VACV, and they can potentially work as bridges promoting the circulation of VACV throughout wild and urban environments and posing a threat to human health [68]. Another study detected the presence of VACV in urban domestic cats in Brazil, reinforcing the possibility of transmission of VACV to humans and veterinary professionals, similar to the cases of CPXV in Europe [67].

Another study showed molecular evidence of OPV circulation in capybaras that inhabit the Lagoa da Pampulha (part of the famous architectural complex of Pampulha in Belo Horizonte, Southeast region of Brazil) and surroundings, raising questions about these rodents in the VACV transmission cycle, as well as the presence of VACV in urban areas [73]. Further studies have also demonstrated VACV circulation in capybaras in the State of São Paulo, confirming the susceptibility of these large rodents to the virus [74]. Finally, the detection of VACV in other wild animals (rodents, marsupials, chiropterans, and cingulates) in the South and Southeast regions of Brazil, outside the context of BV outbreaks, revealed that these animals could potentially act as hosts in the epidemiological chain of VACV in urban environments, and possibly play a role in the transmission to humans [75].

The possibility of disseminating VACV in the urban environment and increasing BV cases should be better investigated to shed more light on the VACV emergence. It is estimated that, due to the discontinuation of smallpox vaccination, a large proportion of the population living in urban environments have never been exposed to OPV [3,76,77]. Moreover, there are more and more immunosuppressed individuals in a society due to chronic and acquired conditions, whose exposure to VACV could evolve into a severe form of infection. Furthermore, the potential establishment of the virus in urban domesticated animals can contribute significantly to the maintenance of an urban cycle [17,27,78,79]. Hence, further studies are necessary to better understand the potential entry of VACV into the urban environment. 

**Figure 3:**
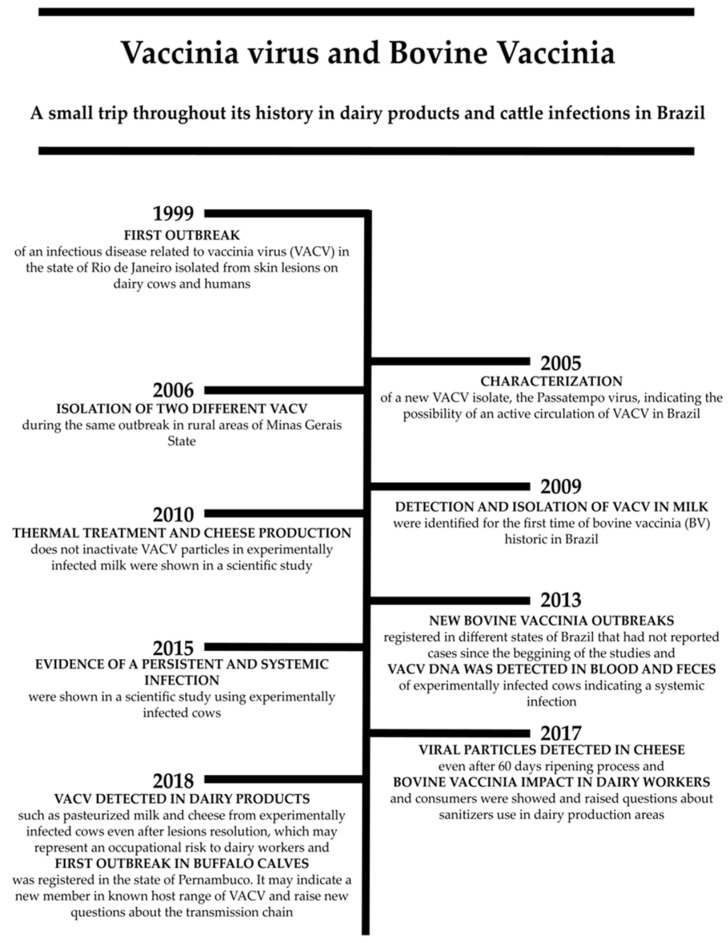
A vertical timeline of the main events regarding the Vaccinia virus occurrence dairy products and cattle in Brazil during 2000–2020.

As BV affects mainly dairy cattle and milkers, several queries regarding the transmission of VACV through milk and dairy products have been raised. Even during scientific conferences in Brazil and worldwide, questions such as “is there any chance someone could get infected with VACV by drinking milk from infected cows?” have emerged. Indeed, studies focused on the relationship between VACV transmission and dairy production have grown significantly over these 20 years of VACV history in Brazil [20,21,22]. Figure 3 displays a timeline with chronological events related to the detection of VACV in dairy products. The first records of the presence of VACV in milk samples from naturally infected cows occurred during 2005–2008. Abrahão and colleagues detected and isolated viral particles on chicken egg chorioallantoic membranes (CAM) and also observed cytopathic effects on embryo fibroblasts (CEFs), in addition to molecular and phylogenetic analysis [80]. Hence, this study became one of the main references to raise questions about milk’s risks as a potential alternative route of VACV transmission.

In the following years, other correlated researches would appear, revealing that VACV viral particles could not be inactivated even after thermal treatments of milk and during cheese processing, both in their production and in their maturation period. The detection of infectious viral particles (from both groups I and II) in milk samples and dairy products confirmed this hypothesis [80,81,82,83,84].

It is noteworthy that serological and molecular evidence was detected in naturally and experimentally infected cows, and dairy products produced from the milk of these animals, raising the discussion about the persistence of systemic infection in these animals [13,84]. In addition to this evidence, Borges et al. demonstrated that even in rural properties with no record of BV outbreaks, dairy cattle could present neutralizing antibodies against VACV [22]. Furthermore, the practice of milking without properly sanitizing cows’ udders can directly affect cattle exposure and milkers [22]. All the studies conducted over the past few years reinforce the possibility that dairy products could work as vehicles for VACV dissemination and potential risks generated to public health [78,80,81,82,83,84].

## 5. The Transversal Impact of BR-VACV

In the last 20 years, milk and dairy products’ production and consumption have grown significantly in Brazil. Data from Brazilian Agricultural Research Corporation (EMBRAPA) [85] have shown that the milk production in Brazil increased 139% between 1990 and 2019. Brazil ranks as fourth among 20 countries regarding total milk production and annual growth rates, being behind India, the United States, and Pakistan [85,86]. The State of Minas Gerais leads the ranking of the milk production, followed by Paraná and Rio Grande do Sul [85,86]. 

The tradition in milk production and its derivatives is a striking feature of Minas Gerais State, significantly impacting the Brazilian economy [85]. Historically, Minas Gerais has a tradition in dairy production and has been primarily affected by BV outbreaks. In addition to dairy farming, the State of Minas Gerais is internationally known for its production of artisanal cheeses prepared by local farms in the countryside, being registered as intangible and cultural heritage, showing great relevance to the State economy, as well as to the country [85,86,87]. Given this traditional feature, dairy products can also be considered as essential assets for tourism purposes. According to Kamimura et al., the State of Minas Gerais has lots of tourist regions and many tourists feel attracted by the artisanal cheese production, learning about the process during scheduled tours [88]. This recognition directly impacts the economy of Minas Gerais. Thus, all issues affecting dairy production in the local farms greatly affect the local economy and national.

The demonstration that these products can act as alternative routes for zoonotic VACV transmission, the demand for monitoring and developing preventive strategies for quality control of both milk and its derivatives are necessary. Furthermore, proper training emphasizing the importance of hygiene measures to milkers focused on milking activities and artisanal cheese production would be essential. Therefore, the awareness and training of milkers and farmers to acceptable dairy practices, the identification of any problems during production, and the supervision and orientation by trained professionals would be critical to result in excellent and safe products. 

The quality control of milk and dairy products through the process of pasteurization is of great importance in preventing the transmission of VACV and other infectious agents, also preventing these products to potentially act as alternative routes of viral dissemination in the rural and also urban environments [17,22,25,87,88]. However, the impact of milk and dairy products as vehicles for VACV spreading is still poorly explored. In regions recognized by the production as artisanal cheese, the burden of VACV maybe even more significant since the artisanal cheeses are essentially made with crude milk (no thermal processes are applied) [89,90,91].

In Brazil, very few laboratories have the knowledge, tools, and capacity for BV diagnostics, despite many outbreaks have been described in the country (especially in the Southeast region). In addition, the increasing danger of potential establishment of VACV urban cycles and consequent outbreaks makes it extremely necessary to invest in research that can elucidate questions about the eco-epidemiological cycle in different areas, the origin of the virus, its natural reservoirs, and new therapies to control the burden of VACV infections. Furthermore, the investment in new, efficient, and rapid tools for VACV and other OPV detection and increasing the diagnostic capacity for other laboratories throughout Brazil would be valuable for the surveillance and monitoring of VACV and future BV outbreaks.

The consolidation of VACV infections in large populations can lead to problems for public health services. The undergraduate courses in Medicine and Nursing at the main public universities that are considered high-quality educational institutions and where most research is conducted in Brazil, sometimes do not include or dedicate a small portion of the teaching load regarding Poxviruses in disciplines related to Microbiology and Virology (Supplementary Table S2). Thus, newly trained health professionals are not being prepared to deal with the burden of VACV occurrence and even with BV outbreaks.

The exclusion of teaching about Poxviruses in many undergraduate courses can be partially explained by the emergence of new viruses and new diseases that affect humans and are more present in the population’s daily lives after the eradication of smallpox, as well as how health authorities still neglect VACV and BV. As a result, professionals do not know about the clinical symptoms characteristic of poxviruses, including VACV infection and its epidemiology. It has not been uncommon for cases in which health professionals offer incorrect treatment, with the false conception that this is another type of infection, which makes the epidemiological mapping of cases of BV in Brazil even more complex [92].

In 20 years, one study was conducted to evaluate the healthcare professionals’ knowledge and perceptions about bovine vaccinia in Brazil and the results showed that 43.1% of healthcare professionals that work in an endemic area have never heard about the disease, which could be attributed to not being well-informed enough to recognize and therefore report clinical cases accurately [92]. The lack of knowledge of health professionals also reflects in the BV notification data across Brazil.

## 6. Where Are We Going with the BV and/or VACV Threat?

Despite the growing number of scientific publications and research on BV and VACV natural circulation in Brazil, many gaps in the knowledge of its epidemiology and natural history have not been filled yet. The sequencing and genetic characterization of new isolates are still needed to assess genetic relatedness and determine relationships among different BR-VACV strains and hosts. The reports of the expansion of BV in Brazil and in other South American countries have not been enough to establish epidemiological surveillance, being BV and VACV still neglected by health authorities. Because most of the research on VACV and BV was focused on describing the outbreaks and its consequences, there are still many gaps to explore in VACV epidemiology, such as its prevalence among milking cattle, other farms and domestic animals, dairy workers, and the general population.

The absence of a unified system and accurate records provided by the healthcare agencies are out of date and do not demonstrate VACV and/or BV epidemiology’s actual situation. It is necessary to develop continuing education practices for all professionals working with VACV and/or BV. Hence, all kind of professionals dealing with VACV emergence, such as medical doctors, nurses, epidemiologists, veterinarians, laboratory workers, as well as public health authorities, people involved in fieldwork, and administrative personnel, could offer greater credibility in the registration of cases and data, thus providing better and efficient preventive actions.

Furthermore, future studies aiming to understand the prevalence of VACV and/or OPV infections in urban populations and monitor the levels of immunity against VACV are necessary. Additionally, routine disease surveillance through physicians and laboratories is also essential to better understand the presence of VACV and the emergence of outbreaks in vulnerable populations, monitoring the associated risk factors to guide better public health practices.

This review aimed to highlight the main findings in these 20 years of history of VACV in Brazil through studies relevant to the understanding of the situation that the virus and the disease are in the country. The lack of investment in research, the unpreparedness of health professionals and agencies to deal with the disease, and the dairy products showing aptitude as alternative routes of infection raise the question of how BV should be treated from this moment on. Thus, the need for further studies is clarified so that all gaps are filled to control and monitor the disease in Brazil.

## Supplementary Materials

The following are available online at https://www.mdpi.com/article/10.3390/pathogens10040406/s1, Table S1: List of hosts and susceptible animals to BR-VACV, and the association to transmission to humans; Table S2: List of the main Universities in Brazil that mention the teaching of Virology and Poxviruses in their undergraduate courses.
