# Supplementary material for: Twenty Years after Bovine Vaccinia in Brazil: Where We Are and Where Are We Going?
Source: Pathogens. 2021 Mar 31;10(4):406. doi: 10.3390/pathogens10040406 (PMC8065508; doi:10.3390/pathogens10040406)
Supplement: Supplementary file 1 [file pathogens-10-00406-s001.zip › Table 1.docx]

**Table S1.** List of hosts and susceptible animals to BR–VACV, and the association to transmission to humans.

|  | Family/Species | Laboratory identification | References |
| --- | --- | --- | --- |
| **Association with human infections*** | *Bovidae* / domestic buffaloes (*Bubalus bubalis*) and cattle/cows (*Bos taurus*) | DNA detection and virus isolation | 11; 12; 14; 15; 29; 30 |
|  | *Hominidae* / Humans (*Homo sapiens*) | DNA detection and virus isolation | 12; 14; 15; 18-21; 29 |
|  | *Muridae* / Inbred-mouses (*Mus musculus*) | DNA detection and virus isolation | 29; 54; 67; 70; 72; 73 |
|  |  |  |  |
| **No association with human infections** | *Cebidae* / Capuchin monkeys (*Sapajus apella*) | DNA detection | 37 |
|  | *Atelidae* / Black-howler monkeys (*Alouatta caraya*) | DNA detection | 37; 76 |
|  | *Didelphidae* / Black-eared possums (*Didelphis aurita*), White-eared possums (*Didelphis albiventris*) and Wooly-cuycas (*Caluromys philander*) | DNA detection | 32; 37; 67; 76 |
|  | *Equidae* / Horses (*Equus ferus caballus*) | DNA detection and virus isolation | 31; 34; 36 |
|  | *Equidae* / Donkeys (*Equus africanus sp.)* and Mules (*Equus mulus*) | DNA detection | 36; 51 |
|  | *Procyonidae* / Ring-tailed coatis (*Nasua nasua*) | DNA detection | 37; 69 |
|  | *Felidae* / Domestic cats (*Felis catus*) | DNA detection | 32; 68 |
|  | *Canidae* / Domestic dogs (*Canis familiaris*) | DNA detection | 32; 69 |
|  | *Chlamyphoridae* / Armadillos (*Euphractus sexcintus*) | DNA detection | 37 |
|  | *Molossidae* / Black-molossus bats (*Molossus rufus*) and Broad-eared bats (*Eumops perotis*) | DNA detection | 76 |
|  | *Cricetidae* / *Oryzomys* spp. | DNA detection and virus isolation | 54; 57 |
|  | *Cricetidae* / Black-footed colilargos (*Oligoryzomys nigripes*), Yellow pygmy rice rats (*Oligoryzomys flavenscens*), Rat-headed rice rats (*Sooretamys angouya*), Vesper mouses (*Calomy*s spp.), Grass mouses (*Akodon* spp.), Hairy-tailed, Bolo Mouses (*Necromys Lasiurus*) and Bush mouses (*Cerradomys subflavus*) | DNA detection | 32; 57; 73; 76 |
|  | *Echimyidae* / Hairy Atlantic spiny rats (*Trinomys setosus*) | DNA detection | 57 |
|  | *Muridae* / Black-mouses (*Rattus rattus*) | DNA detection | 33; 57; 76 |
|  | *Caviidae* / Capybaras (*Hydrochoerus hydrochaeris*) | DNA detection | 74; 75 |

*Transmission to humans already reported in the literature.
