# Supplementary material for: Twenty Years after Bovine Vaccinia in Brazil: Where We Are and Where Are We Going?
Source: Pathogens. 2021 Mar 31;10(4):406. doi: 10.3390/pathogens10040406 (PMC8065508; doi:10.3390/pathogens10040406)
Supplement: Supplementary file 1 [file pathogens-10-00406-s001.zip › Table 2.docx]

**Table S2.** List of the main Universities in Brazil that mention the teaching of Virology and Poxviruses in their undergraduate courses

| **Region of Brazil** | **University** | **Abbreviation** | **Academic program** | **Does it mention the teaching of Virology in the program or pedagogical plan?** | | **Does it mention the teaching of the theme Poxvirus in the program or pedagogical plan?** | |
| --- | --- | --- | --- | --- | --- | --- | --- |
|  | Universidade de Brasília | UnB | Nursing Medical Nursing Medical Nursing Medical Nursing Medical Nursing Medical Nursing Medical Nursing Medical Nursing Medical Nursing Medical Nursing Medical Nursing Medical Nursing Medical Nursing Medical Nursing Medical Nursing Medical Nursing Medical Nursing Medical Nursing Medical Nursing Medical Nursing Medical Nursing Medical Nursing Medical Nursing Medical Nursing Medical Nursing Medical Nursing Medical Nursing  Medical | YES YES YES YES YES YES YES YES YES YES YES YES YES YES YES YES YES YES YES YES YES YES YES YES YES YES YES YES YES YES YES YES  YES YES  YES YES YES YES YES YES YES YES YES YES YES YES YES YES YES YES YES |  |  | NO NO NO NO NO NO NO  NO NO NO NO NO NO NO NO NO NO NO NO NO NO NO NO NO NO NO NO NO NO NO NO NO NO NO NO NO NO NO NO NO NO NO  NO NO NO NO NO NO NO NO NO NO |
|  | Universidade Federal de Goiás | UFG |  |  |  |  |  |
| MIDWEST |  |  |  |  |  |  |  |
|  | Universidade Federal do Mato Grosso | UFMT |  |  |  |  |  |
|  | Universidade Federal do Mato Grosso do Sul | UFMS |  |  |  | YES |  |
|  | Universidade Federal de Alagoas | UFAL |  |  |  |  |  |
|  | Universidade Federal da Bahia | UFBA |  |  |  |  |  |
|  | Universidade Federal do Ceará | UFC |  |  |  |  |  |
|  | Universidade Federal do Maranhão | UFMA |  |  |  |  |  |
| NORTHEAST | Universidade Federal da Paraíba | UFPB |  |  |  |  |  |
|  | Universidade Federal de Pernambuco | UFPE |  |  |  |  |  |
|  | Universidade Federal do Piauí | UFPI |  |  |  |  |  |
|  | Universidade Federal do Rio Grande do Norte | UFRN |  |  |  |  |  |
|  | Universidade Federal de Sergipe | UFS |  |  |  |  |  |
|  | Universidade Federal do Acre | UFAC |  |  |  |  |  |
|  | Universidade Federal do Amapá | UNIFAP |  |  |  |  |  |
|  | Universidade Federal do Amazonas | UFAM |  |  |  |  |  |
| NORTH | Universidade Federal do Pará | UFPA |  |  | NO NO |  |  |
|  | Universidade Federal de Rondônia | UNIR |  |  |  |  |  |
|  | Universidade Federal de Roraima | UFRR |  |  | NO |  |  |
|  | Universidade Federal de Tocantins | UFT |  |  |  |  |  |
|  | Universidade Federal do Espírito Santo | UFES |  |  |  |  |  |
|  | Universidade Federal de Minas Gerais | UFMG |  |  |  |  |  |
| SOUTHEAST |  |  |  |  |  | YES |  |
|  | Universidade Federal de São Paulo | UNIFESP |  |  |  |  |  |
|  | Universidade Federal do Rio de Janeiro | UFRJ |  |  |  |  |  |
|  | Universidade Federal do Paraná | UFPR |  |  |  |  |  |
| SOUTH | Universidade Federal do Rio Grande do Sul | UFRGS |  |  |  |  |  |
|  | Universidade Federal de Santa Catarina | UFSC |  |  |  |  |  |
